# Coupled gyration modes in one-dimensional skyrmion arrays in thin-film nanostrips as new type of information carrier

**DOI:** 10.1038/srep45185

**Published:** 2017-03-22

**Authors:** Junhoe Kim, Jaehak Yang, Young-Jun Cho, Bosung Kim, Sang-Koog Kim

**Affiliations:** 1 National Creative Research Initiative Center for Spin Dynamics and Spin-Wave Devices, Nanospinics Laboratory, Research Institute of Advanced Materials, Department of Materials Science and Engineering, Seoul National University, Seoul 151-744, Republic of Korea

## Abstract

We report on a micromagnetic numerical simulation study of dynamic coupling between neighboring skyrmions periodically arranged in narrow-width nanostrips. We explored the coupled gyration modes and their characteristic dispersions in terms of the interdistance between the neighboring skyrmions. The application of perpendicular magnetic fields allows for the control and modification of the dispersion of the coupled gyration modes. The coupled gyration modes of individual skyrmions might provide a new type of information carrier in narrow-width straight and curved nanostrips, as driven by magnetic interactions in such continuous thin films.

Topologically stable magnetic skyrmions^1^,^2^ have been observed in both bulk magnetic materials of non-centrosymmetry^3^,^4^ and magnetic thin films of broken inversion symmetry at hetero-interfaces of large spin-orbit coupling^5^,^6^,^7^. It is well known that the Dzyaloshinskii-Moriya interaction (DMI)^8^,^9^ plays a crucial role in stabilizing the unique spin textures of skyrmions in bulk and thin-film materials^3^,^4^,^5^,^6^,^7^. The characteristic features of skyrmions, including nano-scale size, topological stability, and ultra-low threshold current density necessary for their motions, make them promising potential candidates for information-storage and -processing device applications^6^,^10^,^11^,^12^,^13^,^14^,^15^. Moreover, recent observations of room-temperature magnetic skyrmions have attracted increasing interest for the fundamental and technological implications^16^,^17^,^18^, For example, one dimensional (1D) skyrmion arrays in FeGe nanostrips were found experimentally^19^.

Additionally to such exotic spin textures, fundamental dynamic modes of skyrmion crystals were found theoretically by M. Mochizuki^20^ and also experimentally by Y. Onose *et al*.^21^. Both studies revealed the existence of skyrmion-core gyration modes of either the clockwise (CW) or counter-clockwise (CCW) rotation sense, as excited by in-plane ac magnetic fields^20^,^21^, as well as another breathing mode^20^,^21^ excited by out-of-plane ac magnetic fields. Such internal dynamic modes were also found in single skyrmions in infinite films^22^ or in confined geometries^23^,^24^. Furthermore, collective excitations in 1D chains of single-skyrmion nanodisks^25^, and propagations of spin-waves and their dispersion characteristics in 1D periodic skyrmion lattices^26^ have been studied.

Whereas the above studies focused on the fundamental dynamic modes in single skyrmions^22^,^23^,^24^ and 1D skyrmion arrays^25^,^26^, coupled gyration modes in 1D skyrmion lattices in thin-film nanostrips have yet to receive much attention in terms of the implications for information carriage in straight and curved nanostrips without the direct motion of skyrmion positions. In the current work, therefore we not only explored the gyration modes of two, five and more coupled skyrmions in continuous thin-film nanostrips but also studied the effects of skyrmion-to-skyrmion interdistance and perpendicular magnetic fields on those modes. Those characteristic behaviors were examined from the aspect of their potential applications as spin-based information carriers.

## Results

### Intrinsic dynamic modes of single skyrmion in nano-square dot

**Figure 1:**
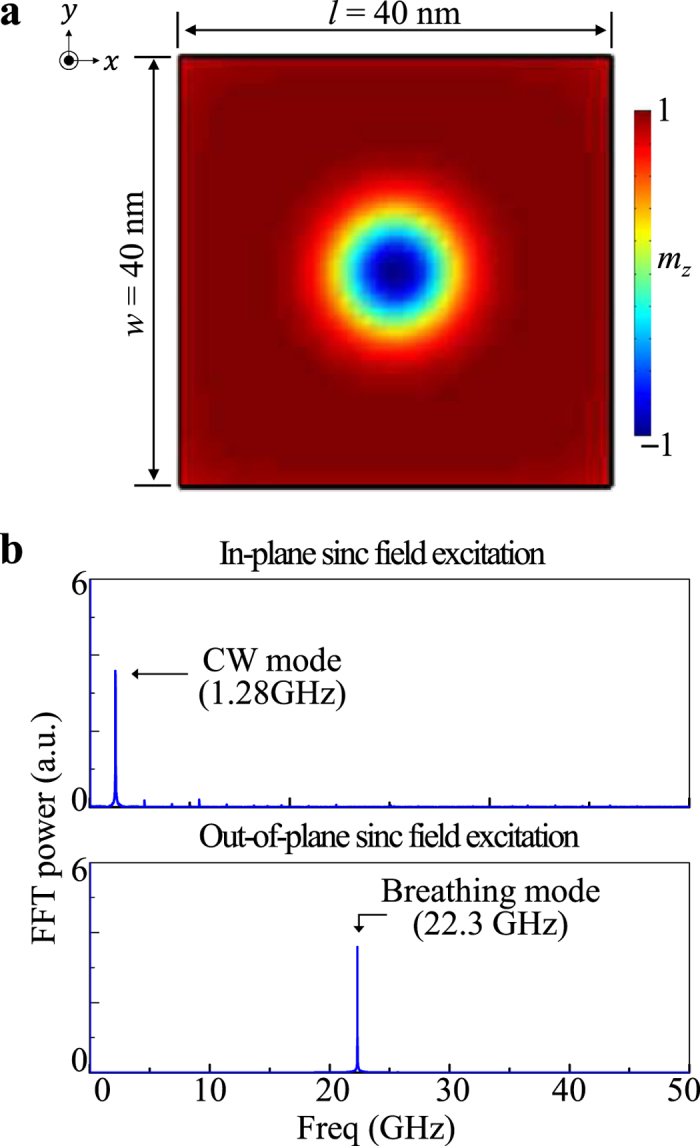
Intrinsic modes of a single skyrmion in a nano-square dot. (**a**) Single skyrmion in perpendicularly magnetized nano-square dot of indicated dimensions. The colors correspond to the out-of-plane magnetization components *m*_z_ = M_z_/M_s_. (**b**) FFT power spectra in frequency domain, as obtained from fast Fourier transform (FFT) of temporal oscillations of *y* position of skyrmion-core motion excited by in-plane sinc field (top) and oscillation of *m*_*z*_ = *M*_*z*_*/M*_*s*_ averaged over entire nano-square dot excited by out-of-plane sinc field (see the text for the form of sinc field used).

In the simulation, first, to determine the intrinsic dynamic modes of an isolated skyrmion, we employed, as shown in Fig. 1(a), a single skyrmion of downward core orientation in a 40 nm-wide square dot of thickness of *h* = 1 nm. Sinc-function fields, denoted as *H* = *H*_0_ sin[*ω*_*H*_(*t* − *t*_0_)]/[*ω*_*H*_(*t* − *t*_0_)] with *H*_0_ = 10 Oe, *ω*_*H*_ = 2*π* × 50 GHz, and *t*_0_ = 1 ns were applied along the *y* or *x* (in-plane^25^) and *z* (out-of-plane^23^,^25^) axis during a time period of *t = *100 ns. The main features obtained by applying the fields along the *y* and *x* axes are the same. Figure 1(b) shows the spectra obtained from the fast Fourier transform (FFT) of the temporal oscillations of the *y* component of the skyrmion-core position under an in-plane ac magnetic field (upper panel), as well as the temporal oscillations of the *m*_*z*_* = M*_*z*_*/M*_*s*_ component averaged over the entire area of the square dot under an out-of-plane ac field (bottom). The skyrmion-core positions were obtained with reference to the guiding center R = (*X, Y*) of the skyrmion, defined as 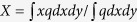 and 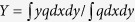, where *q* = (1/4*π)**m*** · (∂_*x*_***m*** × ∂_*y*_***m***) is the topological charge density^27^. As can be seen, two distinct peaks were found at 1.28 and 22.3 GHz, which correspond to the eigenfrequencies of the CW gyrotropic and breathing modes, respectively. These two single-skyrmion modes are consistent with the CW gyrotropic and breathing modes in a confined disk, as found in earlier work^23^,^25^. The gyration mode of an isolated skyrmion also is similar to the well-known gyration mode of a single vortex in soft nanodots.

### Coupled modes of two skyrmions

**Figure 2:**
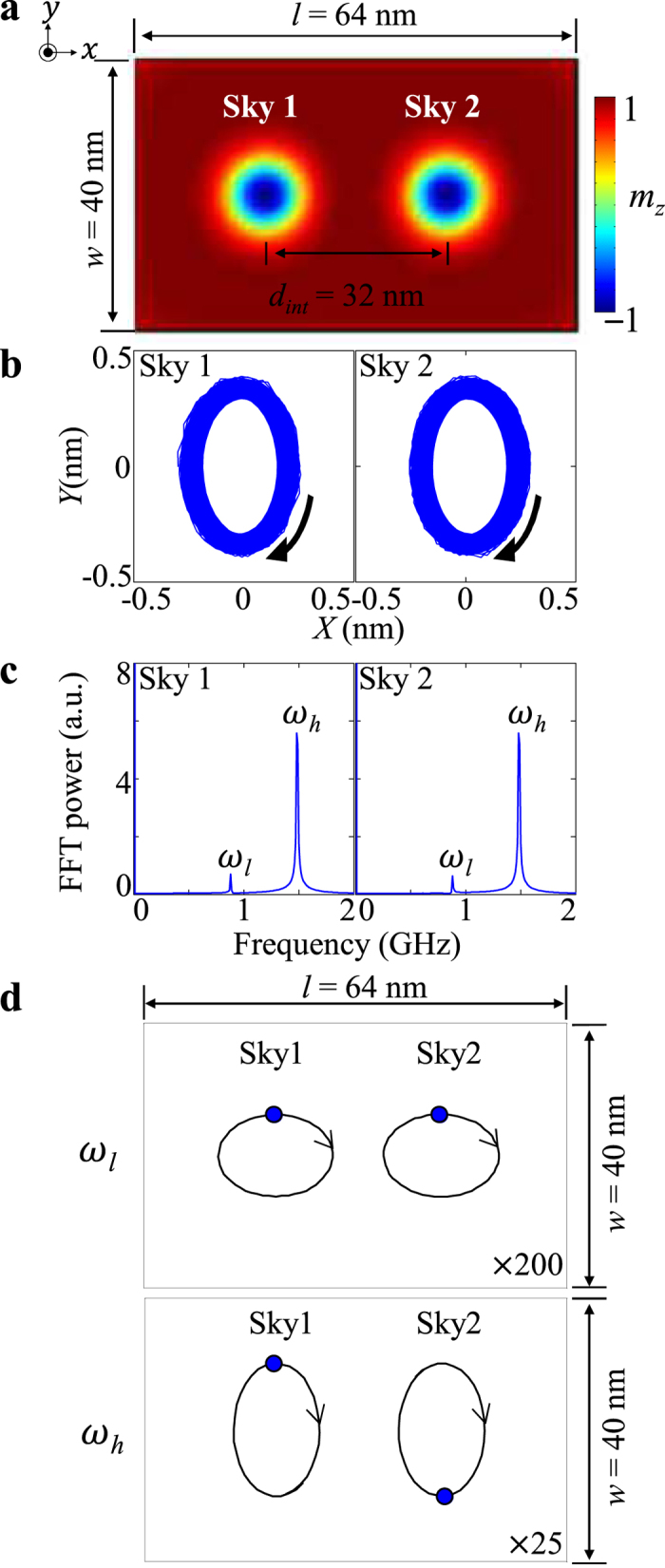
Normal-mode representation of two coupled skyrmions in a rectangular dot. (**a**) Two coupled skyrmions in rectangular dot of indicated dimensions. (**b**) Trajectories of skyrmion-core motions in period of *t* = 0–100 ns. (**c**) FFT power spectra obtained from FFTs of *y* components of two core-position vectors from their own center positions. (**d**) Trajectories of two core motions in one cycle period (2π/*ω*) of the gyration for each mode. The blue dots on the trajectory curves represent the positions of the individual cores. The trajectories of the cores’ motions are magnified for clear comparison, with the magnification power indicated at the right bottom.

In previous work, it was reported that the gyrations of single vortices are coupled to result in collective modes in dipolar-coupled vortices or exchange-and-dipolar-coupled vortex-antivortex systems. From the technological point of view, such coupled modes can be used as information carriers, as already reported in refs 28, 29, 30, 31, 32. Therefore, in the present study, we also took into account dynamic coupling between two skyrmions, both of which are magnetically coupled, as shown in Fig. 2(a). To excite the coupled gyration modes in the two skyrmions, we applied a pulse magnetic field of 700 Oe strength and 10 ns duration in the +*x* direction, locally only to the first skyrmion (noted as Sky1) in order to displace its initial core center to ~1 nm in the –*x* direction. After switching off the local field, we traced the trajectories of both skyrmion centers under free relaxation. The simulation results were obtained up to 200 ns after the field was turned off. The trajectories of the individual core-position vectors **X** = (*X, Y*) are plotted in Fig. 2(b) along with their power spectra in the frequency domain as obtained from the FFTs of the *y* component of the individual core oscillations, as shown in Fig. 2(c). For both skyrmions, two peaks, denoted as *ω*_*l*_ and *ω*_*h*_ that correspond to 2*π* × 0.88 and 2*π* × 1.48 GHz, respectively, were observed. From the inverse FFTs of each peak (mode) for each skyrmion, we obtained the spatial correlation between the two core motions for the *ω*_*l*_ (top) and *ω*_*h*_ (bottom) modes, as shown in Fig. 2(d). For the *ω*_*l*_ mode, the two cores move in phase, while for the *ω*_*h*_ mode, the two cores move in antiphase. For the skyrmions, both having downward cores, the core motion is in the CW rotation sense, which is the same as that of the vortex-state disks having downward cores. Also, the elliptical trajectories of both skyrmion cores’ motions were observed. For the *ω*_*l*_ mode, the major axis of the elliptical shape is along the *x* (bonding) axis, while for the *ω*_*h*_ mode, it is along the *y* axis. The relative magnitude of the trajectories for the *ω*_*l*_ and *ω*_*h*_ modes varies with the excitation field, yielding the overall shape of each core trajectory through the superposition of the two modes, as shown in Supplementary Material Fig. S1. Such different core-trajectory shapes are due to the asymmetry of the skyrmion-skyrmion coupling between the *x*- and *y*- axes.

As reported in refs 33, 34, 35, in the case of two dipolar-coupled vortices in physically separated disks, the dipolar interaction between the two vortex-state disks gives rise to the breaking of the radial symmetry of the potential well of each vortex-state disk. Therefore, the total potential energy of the interacting vortex-state disks can be given as 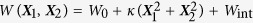, where *W*_0_ is the potential energy for X_1,2_ = (0, 0), the second term is that for the shifted cores with the identical stiffness coefficient *κ* for the isolated disks, and *W*_int_ is the dipolar-coupling energy, which can be obtained from the magnetostatic energy between the side surfaces of two disks on the basis of the rigid vortex model^33^,^34^. On the other hand, in a two-skyrmion system where the skyrmions are magnetically coupled in continuous thin films, the coupled gyration modes and their splitting are also explained by the symmetry breaking of the potential energy of the isolated skyrmions. However, since the individual skyrmions arrayed in continued thin films are directly coupled by the combination of the DMI, perpendicular anisotropy, and exchange and dipolar interactions, we could not extract the *W*_int_ term from the total potential energy of a given entire system. Here, then, we are rather interested in the magnetic-energy term that results dominantly in direct coupling between the neighboring skyrmions. As shown in Supplementary Materials Fig. S2, we calculated the particulars of the exchange, DMI, dipolar, and perpendicular anisotropy energies versus time with respect to the corresponding energies of the initial ground state, and carried out the FFTs of their temporal oscillations during coupled gyration motions. We found that (1) the DMI is the largest energy variation during the coupled dynamic motion, while the dipolar energy variation is relatively small compared with the other energy terms; (2) the frequencies of the temporal oscillations of the individual energies, obtained from their FFTs, are exactly equal to the value of the higher ω_*h*_ mode of the coupled gyrations; (3) the out-of-phase motion between the neighboring skyrmion cores results in such large variations of individual energies, while the in-phase motion does not much change those energy terms (we confirmed that the ω_*l*_ mode peaks are absent from the FFT power spectra of the individual energy oscillations). This result implies that the DMI can play the largest role in coupled-skyrmion gyration motions, though we could not distinguish the *W*_int_ term from the total energy. Additionally, we performed simulations on two exchange-decoupled skyrmions in physically separated skyrmions. We found that the coupling between the cores in exchange-decoupled skyrmions is very weak, therefore, the frequency splitting that represents coupling does not appear and the individual energy oscillations exhibit very weak coupling in the geometry.

### Coupled modes in five-skyrmion array in nanostrip

**Figure 3:**
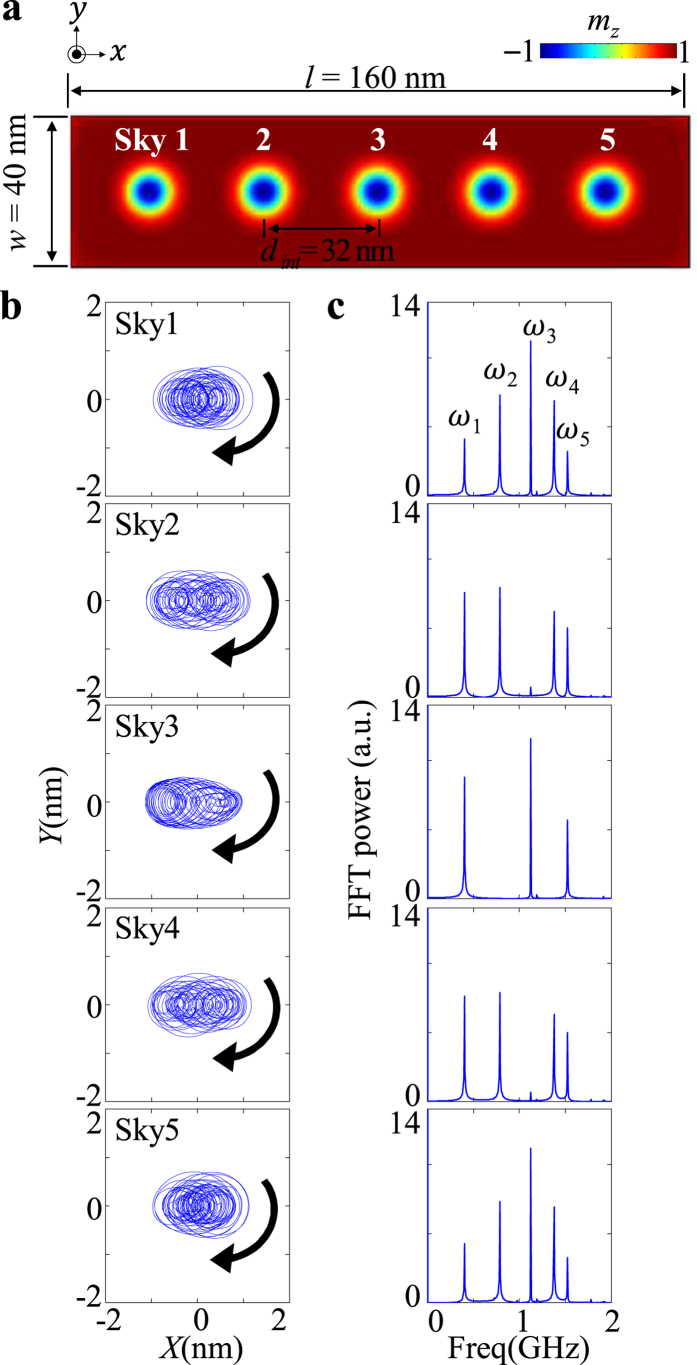
Normal-mode representation of five coupled skyrmions in a nanostrip. (**a**) Five-skyrmion chain in perpendicularly magnetized nanostrip. (**b**) Trajectories of gyration motions of individual cores of five skyrmions and (**c**) their FFT spectra. The five distinct peaks are denoted as the individual mode *ω*_*i*_ peaks (*i* = 1, 2, 3, 4, and 5).

On the basis of the above results, we extended our simulations to a five-skyrmion chain in a rectangular-shape nanostrip [Fig. 3(a)]. We excited the aforementioned coupled gyration modes in the given system by applying a 10- ns-pulse magnetic field of *H*_x_ = 700 Oe only to the first skyrmion (left end), in the same way as earlier. After the field was turned off, the signal of the gyrations excited from the first skyrmion core propagated to the next skyrmion and reached the 5^th^ skyrmion at the other end under free relaxation. Individual core trajectories are complex, due to the superposition of the individual intrinsic coupled modes’ core motions as well as the fact that their potential energies along the *x* and *y* axes are asymmetric, as shown in Fig. 3(b)^30^. Here, we stress that all the core displacements are extremely small, e.g., within ~1 nm. To better understand the observed complex core trajectories (as superposed by the five different modes), we also plotted, in Fig. 3(c), the frequency spectra for the individual core motions. The five distinct peaks corresponding to the five internal modes are denoted as *ω*_*i*_ (*i* = 1, 2, 3, 4, 5). For each skyrmion core, the five peaks in the frequency domain are at ω/2π = 0.40, 0.79, 1.13, 1.38, and 1.53 GHz. For each skyrmion core, the five peaks are located at the same position, though the FFT powers, from the first skyrmion through the fifth, are contrasting. For the first and fifth skyrmions, all of the five peaks appear. By contrast, for the 2nd and 4^th^ skyrmions, the *ω*_*3*_ peak is absent, while for the 3rd skyrmion, the *ω*_*2*_and *ω*_*4*_ peaks disappear. These spectra together represent the characteristic intrinsic modes of collective motions for the given whole system.

**Figure 4:**
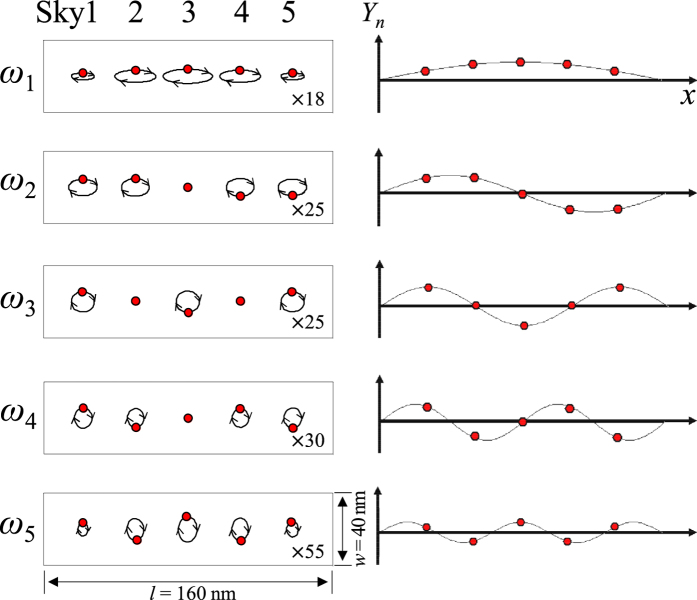
Spatial correlation of the five cores’ gyration motions for each mode. The red dots indicate the core positions of the individual skyrmions. The trajectories are magnified for clear comparison, and the magnifications differ for the individual modes. The corresponding profile of the *y* components is indicated by the solid line in the right panel.

To interpret the complex core motions for each of the five different *ω*_*i*_ modes, we formulated inverse FFTs of the individual cores’ positions. The resultant trajectories of the orbiting cores and the corresponding *y*-component profiles for each mode are illustrated in Fig. 4. On the basis of a fixed boundary condition^28^, the wave vector of the allowed modes is expressed as *k* = *m* · *π*/[(*N* + 1)*d*_int_)], with *N* the number of skyrmions in a nanostrip, *d*_*int*_ the interdistance between the neighboring skyrmions, and *m* a positive integer subject to the constraint *m* ≤ *N*. Thus, the discrete five modes’ *k* values of collective skyrmion-core gyration are given as *k*_*m*_ = *mπ*/6*d*_int_, where *m* = 1, 2, 3, 4, 5, indicating each mode. The collective motions of the individual five skyrmion cores show unique standing-wave forms of different wavelengths, 2*π*/*k*_*m*_. The orbiting radii of the five cores are symmetric with respect to the center of the whole system (i.e., about the 3rd skyrmion) and are also completely pinned at the imaginary positions, 7 nm from both ends [see refs 28, 29, 30]. For the lowest mode, *ω*_1_, all of the cores gyrate in-phase. As the wavelength of those standing waves decreases, the relative phases between the nearest-neighboring cores are increased, and thus, the standing-wave nodes appear.

### Dispersion in 1D skyrmion lattice in nanostrip

**Figure 5:**
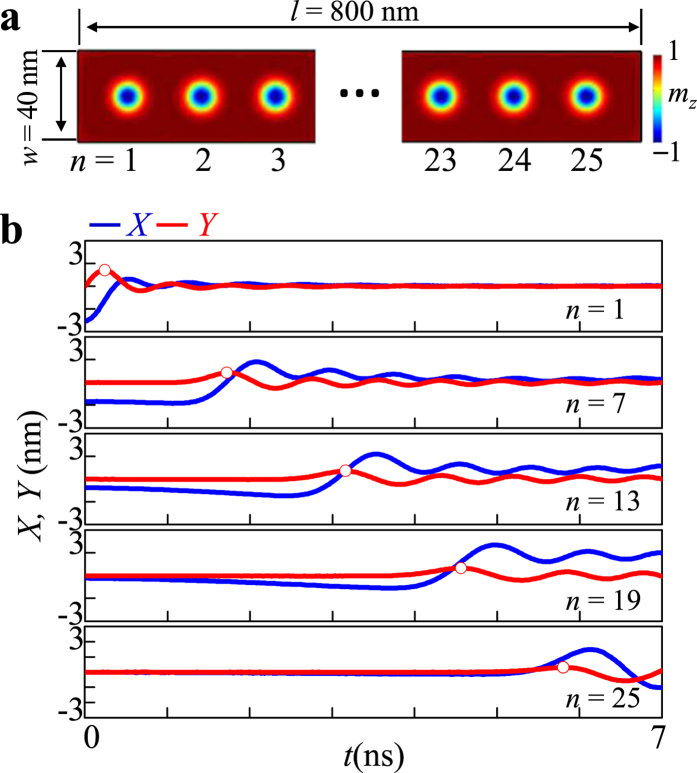
Propagation of excited skyrmion gyrations through 1D skyrmion array. (**a**) 1D skyrmion array in nanostrip of indicated dimensions comprising 25 skyrmions. (**b**) Oscillatory *x* (blue) and *y* (red) components of the displacement of each skyrmion-core position in the *n*th disk. The white dots represent the positions of the first wave packets.

In order to examine the dispersions of such collective motions in 1D coupled finite-number skyrmions, we conducted further simulations of a model of longer 1D chains consisting of *N* = 25 skyrmions in a nanostrip of the following dimensions: length *l* = 800 nm, width *w* = 40 nm, thickness *h* = 1 nm (see Fig. 5(a)). The first skyrmion core was shifted and then allowed to relax in the same way as earlier. Figure 5(b) provides the temporal oscillations of the *x* and *y* components of the individual skyrmion cores. The excited gyration of the first skyrmion core propagated well along the whole nanostrip, as evidenced by the 1st wave-packet propagation through the entire skyrmion chain. The speed of the gyration-signal propagation was estimated, from the 1^st^ wave packets denoted as white dots, to be 135 m/s on average. This speed was more than two times faster than that of vortex-gyration-signal propagation in 1D vortex-state disk arrays^29^ and five times faster than that of edge-to-edge connected skyrmion disk arrays^25^. Faster signal propagation in our geometry is due to the enhanced coupling between the cores in continuous thin film nanostrips by various magnetic interactions.

**Figure 6:**
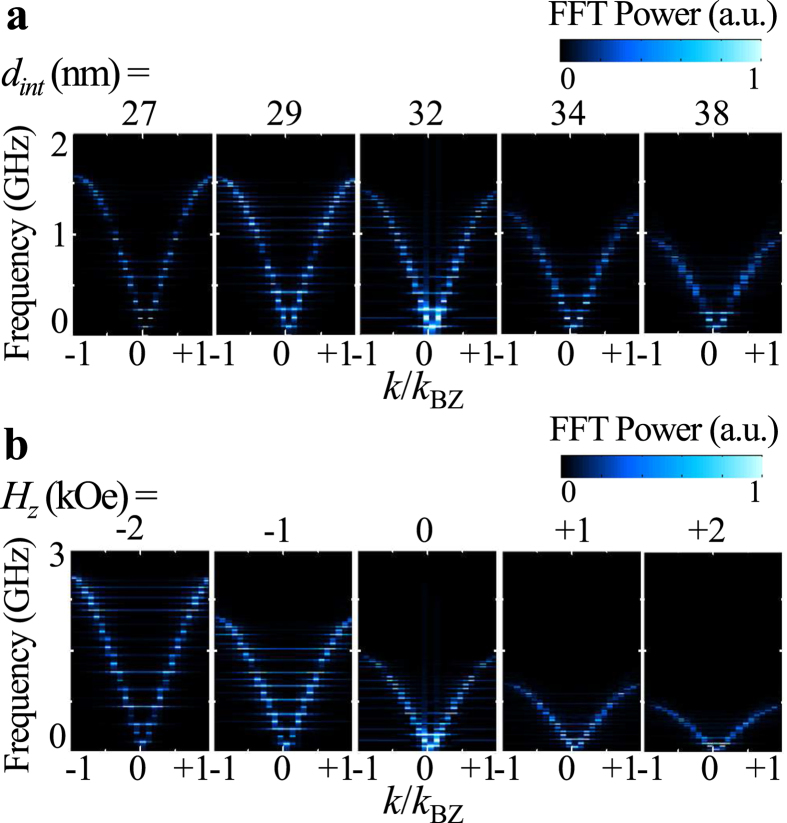
Dependences of the dispersion of 1D skyrmion arrays on interdistance *d*_int_ and perpendicular magnetic field *H*_*z*_. (**a**) Dispersion curves of 1D skyrmion chains for different interdistances, e.g., *d*_*int*_ = 27, 29, 32, 34, 38 nm. (**b**) Dispersion curves of 1D skyrmion chains of 25 skyrmions (*d*_*int*_ = 32 nm) for different perpendicular fields, e.g., *H*_z_ = −2, −1, 0, 1, 2 kOe.

From the FFTs of the temporal oscillations of the *y* components of the individual cores’ position vectors, we also obtained dispersion relations in the reduced zone scheme, as shown in Fig. 6. As is evident in the dispersion curves, the intensities of the modes with positive group velocities are stronger than those with negative group velocities, because the gyration signal was excited from the left end and then was allowed to propagate toward the +*x* direction; the overall shape of dispersion was concave up (that is, the frequency was lowest at *k* = 0 and highest at *k* = *k*_BZ_ = π/*d*_int_); at *k* = 0, all of the cores move together coherently, while at *k* = *k*_BZ,_ they act as the nodes of the standing wave. Such collective dynamic motions are governed by the combination of the various types of magnetic interaction energies.

### Dependences of the dispersion of 1D skyrmion chains on *d*_int_ and *H*_*z*_

**Figure 7:**
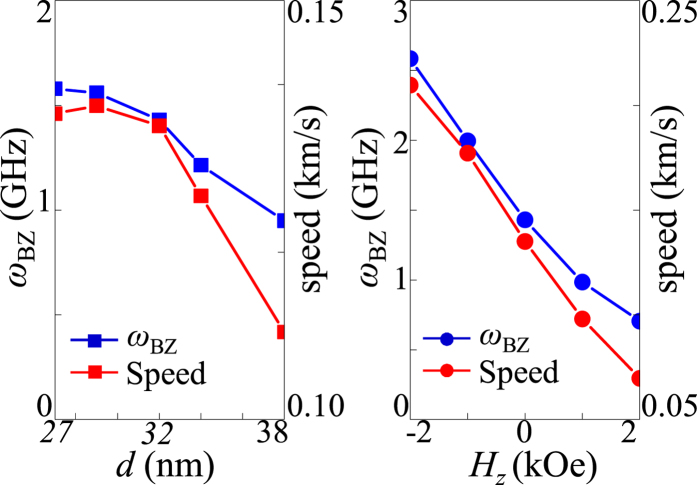
Angular frequency *ω*_BZ_ at *k* = *k*_BZ_, and propagation speed of coupled gyrations versus *d*_*int*_ (left panel) and *H*_z_ (right). The gyration propagation speeds are estimated from the motions of the first wave packets in the chains.

Next, in order to examine the dependence of the band structure of 1D skyrmion lattices on *d*_*int*_, we varied the skyrmion number *N* from 21 to 29 for the given dimensions of the nanostrip, as shown in Fig. 6(a). In the results, stable skyrmion lattices were obtained from *d*_*int*_ = 38 to 27 nm, according to *N*. The contrasting band structures for the different *d*_*int*_ values are shown in Fig. 6(a). It can be seen that as *d*_*int*_ decreases, the band width Δ*ω* and the angular frequency *ω*_BZ_ at *k* = *k*_BZ_ increase (see Fig. 7, left). This increase in Δ*ω* and *ω*_BZ_ with decreasing *d*_*int*_ can be explained by the variation of the magnetic energy density with *d*_*int*_ in the corresponding ground states. As shown in Supplementary Fig. S3, whereas the dipolar and perpendicular anisotropy energy densities do not much vary with *d*_*int*_, the exchange energy density increases remarkably and the DMI energy density decreases with decreasing *d*_*int*_. The increase of the total energy density with decreasing *d*_*int*_ would result in the increase in Δ*ω* and *ω*_BZ_ with decreasing *d*_*int*_.

On the other hand, in additional simulations applying perpendicular magnetic fields of different strengths *H*_z_ = +2, +1, −1 and −2 kOe, we also demonstrated an external control of the band structure of skyrmion lattices. The number of skyrmions in the nanostrip was set as *N* = 25 (i.e., *d*_*int*_ = 32 nm). Figure 6(b) compares the dispersion curves for indicated different *H*_z_ values, showing clearly that the bandwidths of the resultant band structures decrease with increasing *H*_z_. As seen in Fig. 7’s *ω*_BZ_ versus *H*_*z*_ plot, the *ω*_BZ_ also decreases linearly with increasing *H*_*z*_. The application of the *H*_z_ field modifies the magnetization profiles of each of the skyrmions’ cores, as shown in Supplementary Materials Fig. 4. That is, the skyrmion shrinks with the positive external magnetic fields and expands with the negative external magnetic fields. Accordingly, the eigenfreqeuncies *ω*_0_ of the skyrmion’s CW rotation vary with *H*_z_^20^,^36^,^37^. Based on further micromagnetic simulations, we also confirmed that the CW mode’s eigenfrequency of a single skyrmion changes linearly with *H*_z_ (see Supplementary Fig. 5) Therefore, the linear dependences of *ω*_BZ_ on *H*_z_ are mostly associated with the variation of the *ω*_0_ of the isolated skyrmions. In fact, the *ω*_BZ_ value corresponds to the *ω*_0_ of isolated skyrmions, as shown in Supplementary Fig. 5.

## Discussion

From a technological point of view, such gyration-signal propagation in a 1D skyrmion array can be used as a new type of information carrier. Figure 7 shows the gyration-signal propagation speed estimated from the displacement of individual cores from their center positions for different values of *d*_*int*_ (left column) and *H*_z_ (right). The resultant propagation speeds generally follow the dependence of *ω*_BZ_ on *d*_*int*_ (left column) and *H*_z_ (right). The above results can be promising for potential signal-processing applications, because of the following advantages. First, 1D periodic skyrmion lattices at room temperature can be obtained in nanoscale-width nanostrips. Second, the creation or annihilation of single skyrmions as well as the periodicity of skyrmion lattices can be manipulated using various methods including the application of a magnetic probe tip or by control of either the frequency of the magnetic field or the spin-polarized current pulse in nanotracks of different width^7^,^38^. Third, the propagation speed is controllable with an externally applied perpendicular field. Forth, the most important benefit of using the skyrmion gyration modes as information carriers is the fact that skyrmion gyrations can be excited with extremely low power consumption (e.g., an ac resonant field of a few tens of Oe or less^20^, or currents^11^ on the order of ~10^6^ A/m^2^). Finally, such skyrmion-gyration signal propagates even in curved nanostrips (e.g., L or round corners with no edge modifications). We performed additional simulations of a 1D skyrmion array in an L-shaped nanostrip, as shown in Supplementary Fig. S6. It was found that the gyration signal excited from one end propagates well to the other end through the L-shaped nanostrip. In previous work^13^,^39^,^40^, it has been reported that in such curved structure, skyrmion annihilation occurs at the corner edge when the skyrmion motion is driven by currents or spin-waves. Therefore, the method proposed in this work has the merit of being a spin-based logic operation in geometrically complex nano-channel structures. In real applications, lower-damping materials, e.g. Ta/CoFeB/MgO of α = 0.015, can be used to prevent signal loss during gyration-signal propagation^41^. However, the optimization of material parameters and further structural design are necessary to increase the gyration propagation speed for practical applications. Furthermore, in order to realize such a device concept working at room temperature, thermally stable skyrmions are necessary, which should be elucidated in further studies. Here we note that temperature issues (e.g. 1D skyrmion arrays at near-room temperature in FeGe nanostrips^19^, skyrmions at room temperatures in magnetic multilayers^17^,^18^) have been reported recently. Therefore, skyrmion-gyration propagation at elevated temperatures might be achievable through further optimization of material parameters and new structural design.

In summary, we explored the gyration modes of coupled skyrmions and their dispersions in 1D skyrmion lattices. The modes and their characteristic dispersion relations were examined for different skyrmion interdistances and perpendicular magnetic fields externally applied to the nanostrips. Additionally, the controllability of the dispersion curves and skyrmion gyration propagation were demonstrated. Various magnetic interactions between neighboring skyrmions together enhance their coupling, resulting in the propagation of skyrmion gyrations as fast as 100~200 m/s, which value, significantly, are controllable by applied perpendicular fields. This work provides not only a fundamental understanding of the dynamics of coupled skyrmions but also a new type of skyrmion magnonic crystal applicable to future information processing devices.

## Methods

### Micromagnetic simulations

To numerically calculate the dynamic motion of local magnetizations in nanostrips of skyrmion lattices, we employed the Mumax3 code^42^ that utilizes the Landau-Lifshitz-Gilbert (LLG) equation^43^,^44^, 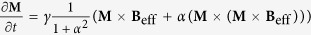, where *γ* is the gyromagnetic ratio, *α* the damping constant, and **B**_eff_ the effective field. For a model system of Co thin films interfaced with heavy metal (Pt) and of perpendicular magnetic anisotropy, we used the following material parameters: *M*_s_ = 580 kA/m, exchange stiffness *A*_*e*x_ = 15 pJ/m, perpendicular anisotropy constant *K*_u_ = 0.8 MJ/m^3^, DMI constant *D* = 3.0 mJ/m^2 ^14. The unit cell was 1.0 × 1.0 × 1.0 nm^3^ in its dimensions. In order to obtain the initial ground states, we assumed periodically arranged Néel-type skyrmion configurations and then allowed them to relax for 100 ns until we obtained their corresponding equilibrium states using a damping constant of α = 0.3. Afterward, the value of *α* = 0.0001 was used to excite coupled dynamics for better spectral resolutions. As illustrated in Supplementary Materials Figs S7 and S8, we also compared the coupled modes for different damping constants, 0.01 versus 0.001. For α = 0.01, the modes’ peaks became broadened and the gyration signals became attenuated, but the peak positions, their dispersion curves, and the gyration propagation speed were not changed relative to those obtained with α = 0.0001.

## Additional Information

**How to cite this article:** Kim, J. *et al*. Coupled gyration modes in one-dimensional skyrmion arrays in thin-film nanostrips as new type of information carrier. *Sci. Rep.* **7**, 45185; doi: 10.1038/srep45185 (2017).

**Publisher's note:** Springer Nature remains neutral with regard to jurisdictional claims in published maps and institutional affiliations.

## Supplementary Material

Supplementary Information
