# Supplementary material for: Coupled gyration modes in one-dimensional skyrmion arrays in thin-film nanostrips as new type of information carrier
Source: Sci Rep. 2017 Mar 22;7:45185. doi: 10.1038/srep45185 (PMC5361123; doi:10.1038/srep45185)
Supplement: Supplementary Information [file srep45185-s1.pdf]

SUPPLEMENTARY INFORMATION

Coupled gyration modes in one-dimensional skyrmion arrays in thin-film nanostrips as new type of information carrier

Junhoe Kim, Jaehak Yang, Young-Jun Cho, Bosung Kim, and Sang-Koog Kim[a)]

National Creative Research Initiative Center for Spin Dynamics and Spin-Wave Devices, Nanospinics Laboratory, Research Institute of Advanced Materials, Department of Materials Science and Engineering, Seoul National University, Seoul 151-744, Republic of Korea

Correspondence and requests for materials should be addressed to S.-K.Kim (sangkoog@snu.ac.kr).

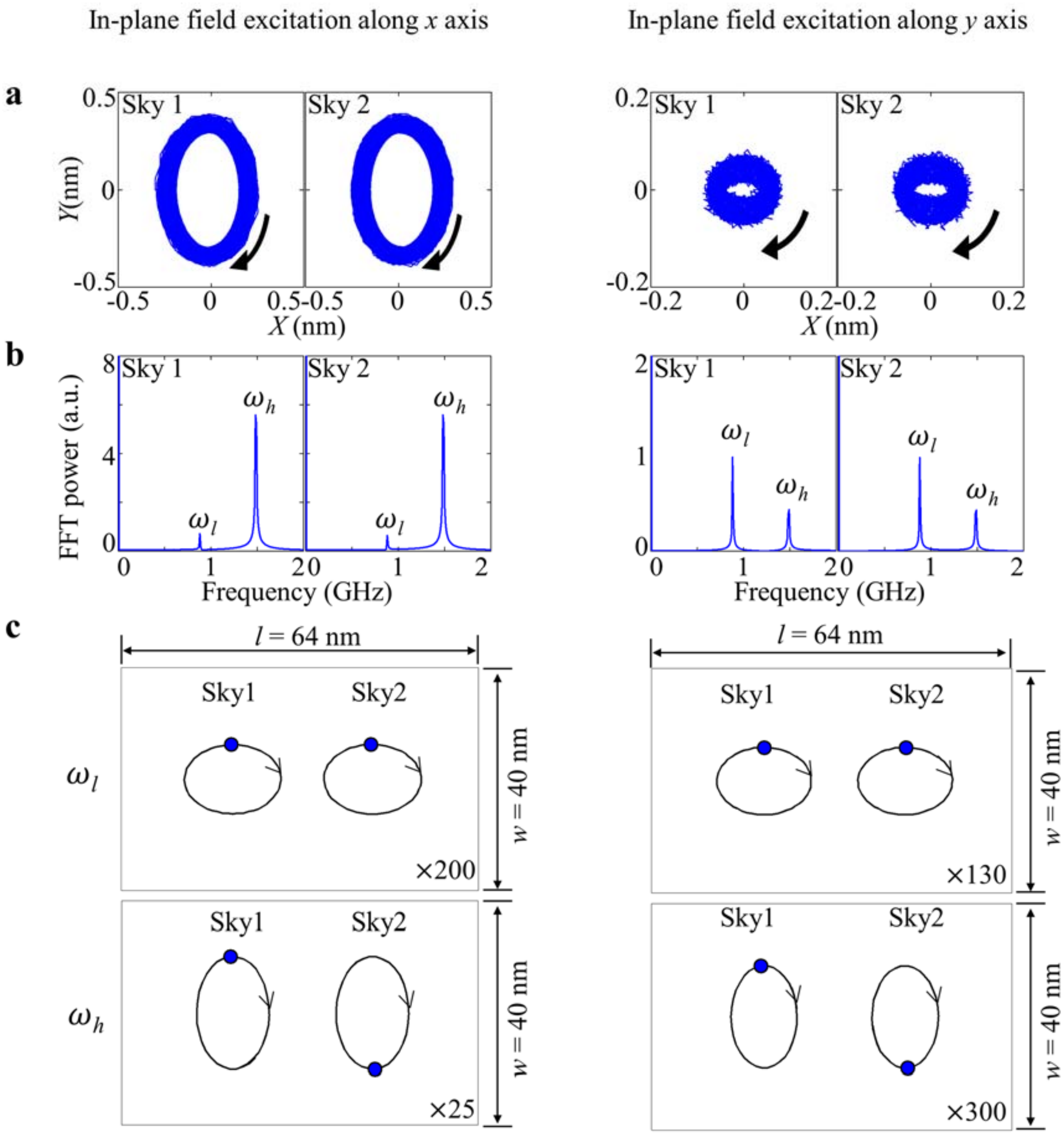

Supplementary Fig. S1. (a) Trajectories of skyrmion-core motions in a period of t = 0-100 ns for the in-plane static fields applied in the +x direction (left column) and the +y direction (right column). (b) FFT power spectra obtained from FFTs of y components of two core-position vectors from their own center positions. (c) Trajectories of two core motions in one cycle gyration for ω_l and ω_h modes, as obtained from inverse FFTs of each peak (mode) for each skyrmion core. The blue dots on the trajectory curves represent the positions of the individual cores at a certain time in one cycle. The trajectories of the cores' motions are magnified for clear comparison, with the magnification power indicated at the right bottom.

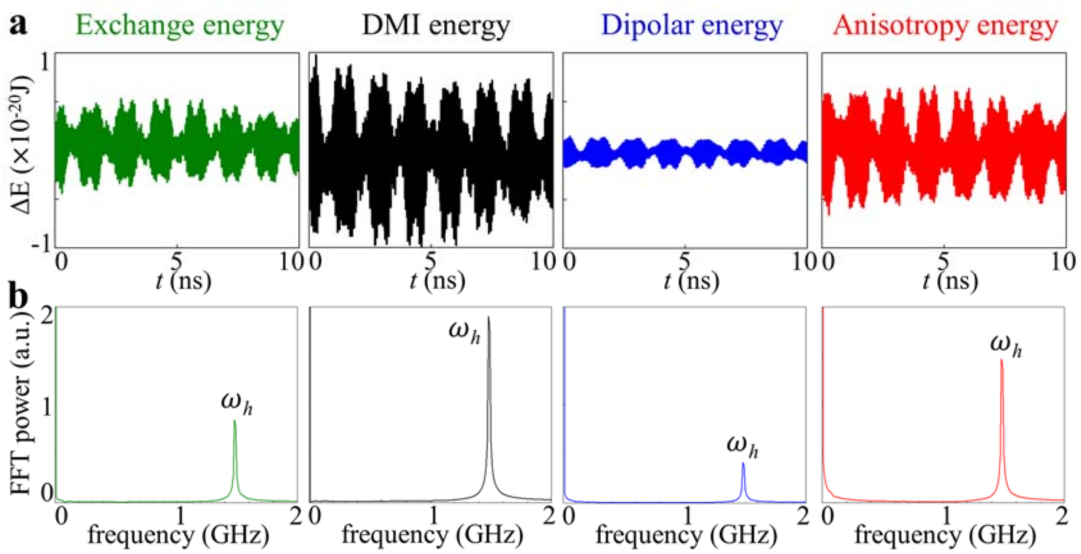

Supplementary Fig. S2. (a) Exchange, DMI, dipolar, and anisotropy energy oscillations during coupled gyration motions in a two-skyrmion system, where the y axis is defined as $\Delta E(t) = E(t) - E(t=0)$. (b) their FFT power spectra. We note that all the peak positions equal to the position of the ω_h mode.

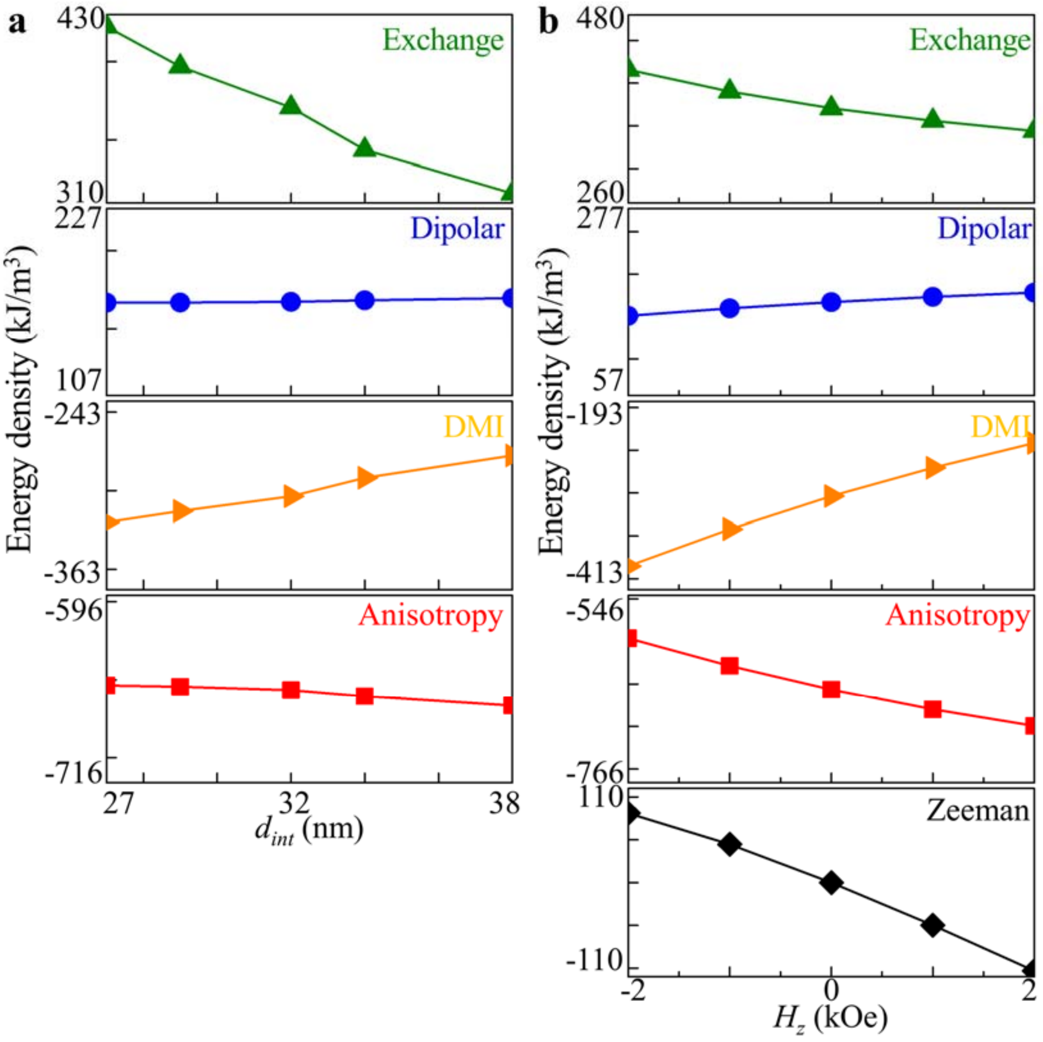

Supplementary Fig. S3. Magnetic energy densities versus (a) d_{int} at H_z= 0 and (b) versus H_z at d_{int}= 32 nm.

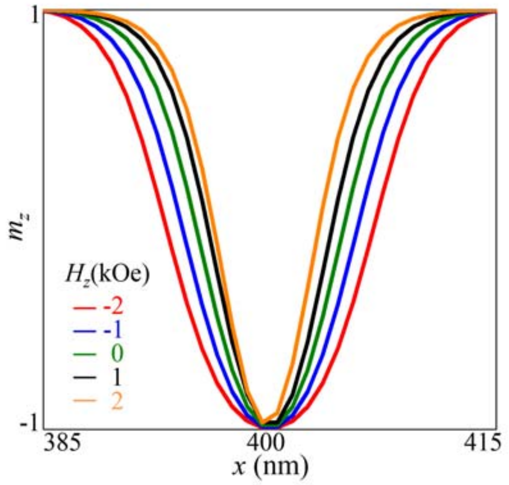

Supplementary Fig. S4. m_z profiles of 13th skyrmion in center region of nanostrip (x = 385–415 nm) at y = 20 nm for N = 25 (d_{int} = 32 nm) skyrmon arrays for different values of perpendicular magnetic field, H_z

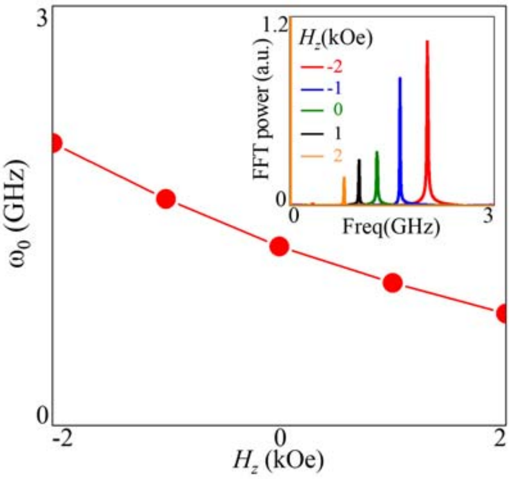

Supplementary Fig. S5. Eigenfrequency ω_0 of CW gyration of single skyrmion in square dot versus H_z. The inset shows the FFT power spectra, as obtained from the FFTs of the temporal oscillations of the y position of the skyrmion-core motion excited by the in-plane sinc field.

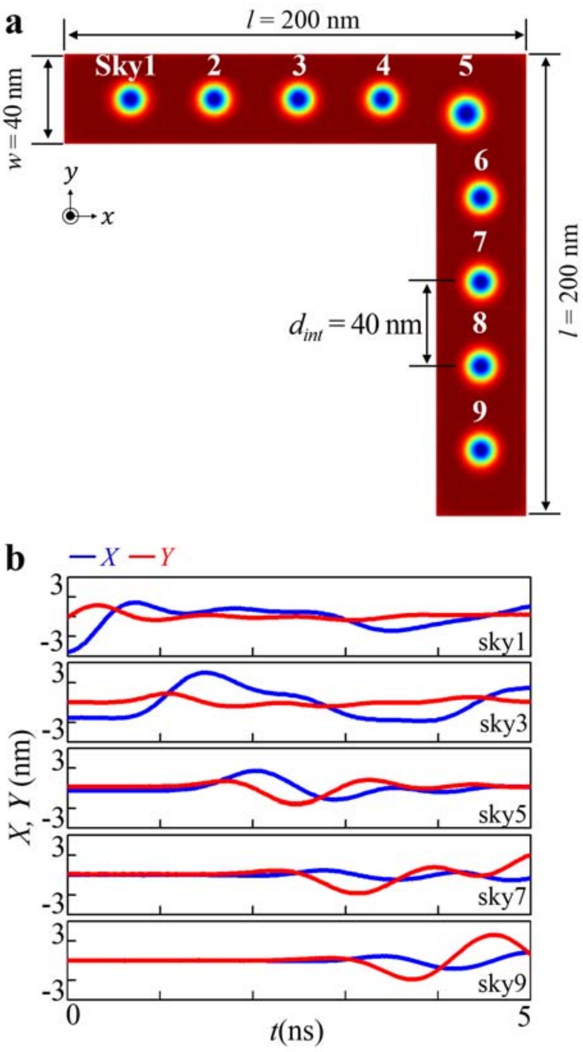

Supplementary Fig. S6. (a) Model geometry of 1D skyrmion array in an L-curved nanostrip. (b) Oscillatory x (blue) and y (red) components of core positions in the nth skyrmion.

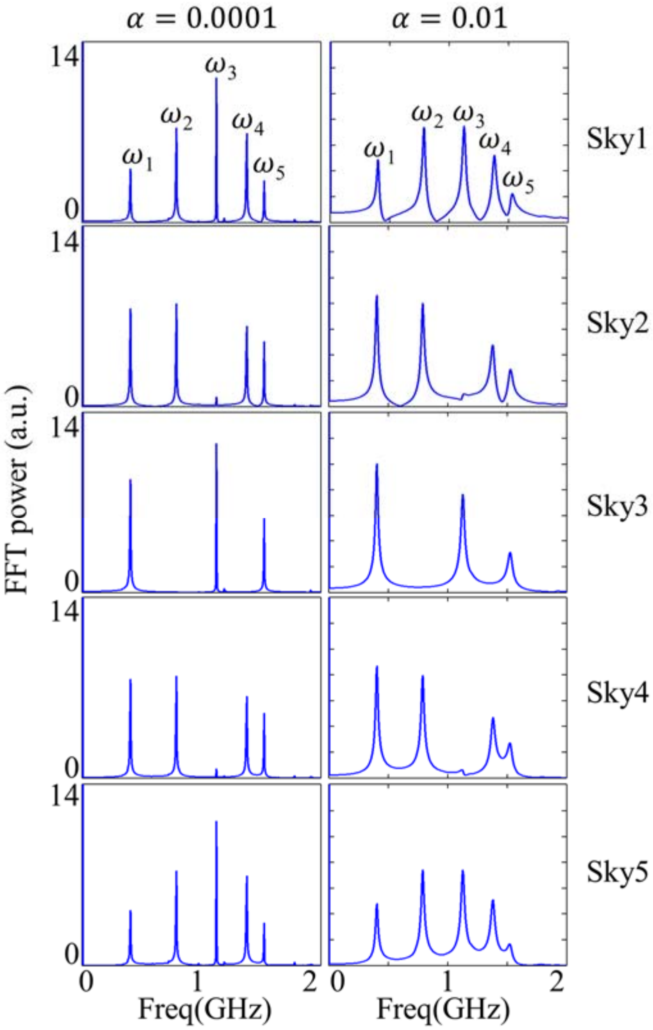

Supplementary Fig. S7. FFT power spectra as obtained from FFTs of the *y* components of individual core-positions in nanostrip for two damping constants, α=0.0001 vs. 0.01.

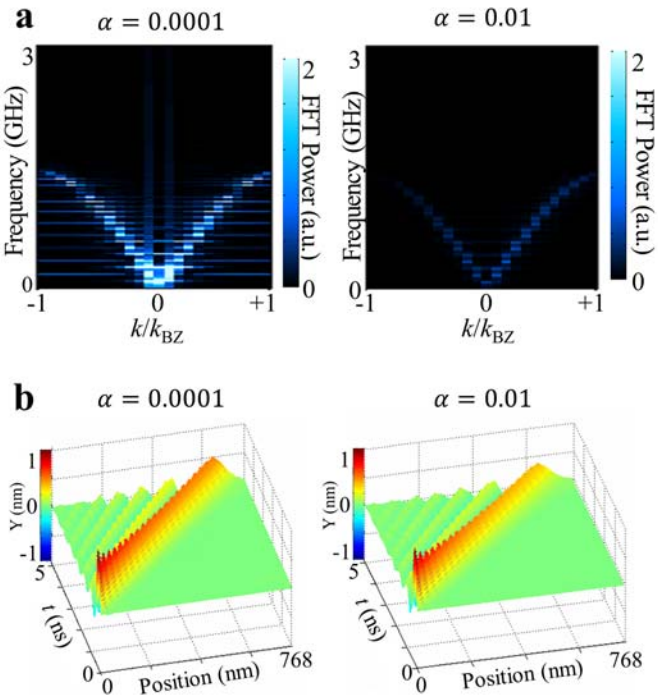

Supplementary Fig. S8. (a) Dispersion curves of 1D skyrmion chains comprised of 25 skyrmions for α =0.0001 and 0.01 and (b) corresponding contour plots of *y* components of individual cores' displacements with respect to time and distance for whole chain. The propagation speed for the different damping constants is the same (~135m/s).
